# The Neuropathic Origin of Edema

**Published:** 1889-12

**Authors:** 


BUFFALO MEDICAL AND SURGICAL JOURNAL

A MONTHLY REVIEW OF MEDICINE AND SURGERY.

*EDITORS:*

THOS. LOTHROP, M. D. - - - W. W. POTTER, M. D.

All communications, whether of a literary or business character, should be addressed to the editors: 284 FRANKLIN STREET, BUFFALO, N. Y.

### Editorial.

### THE NEUROPATHIC ORIGIN OF EDEMA.

SINCE Ranvier's classical experiment on the production of edema, the nervous origin has been undisputed. It will be remembered that he tied the inferior femoral vein, yet edema did not result; but on dividing the sciatic nerve, edema appeared throughout the area of its distribution. These experiments have been repeated by various observers, with identical results. Vulpian believed this to be due to trophic changes in the walls of the vessels. Then Lewaschew made experiments in proof of this theory. He operated on adult dogs. The naked sciatic nerve was exposed, as near as possible to its exit from the pelvis. On one side, he passed through the nerve-trunk a thread saturated with a solution of sodium chloride, so as to produce a more or less acute inflammation. After a short time, he found a corresponding spot, hot and tumefied. After killing the animal, he examined the arteries in the healthy paw, and also in the paw on the operated side.

The large arteries toward the thigh presented nothing appreciable, but in the arteries toward the extremity he found, here and there, spots more or less profoundly inflamed, characterized by a turgescence of the vasa vasorum, and the existence of lymphoidal elements in the external and middle tunic. In some cases the internal tunic shared in the inflammatory proliferation, thus showing endarteritis to be developed more and more as we approach the periphery, where, be it remembered, the vaso-motor action is at its maximum.

We have seen, in practice, similar edemas as the result of nerve wounds.

Weir Mitchell called attention to this, in 1864, in his report on gunshot wounds and other injuries of nerves. He classed these edemas with the other trophic change described by Paget under the name of glossy skin.

In our own practice, we have recently seen edema of the arm in a case of brachial neuralgia, due, probably, to neuritis. As the pain subsided, the edema disappeared.

These edemas may subside and reappear as the nerve lesion is improved or is newly aggravated.

Matthew and Weil, in a joint paper, described three forms which the edema presents:

In one case it is found to be white, soft, and easily depressed, and not painful. Again, it may be hard, sensitive on pressure, and gives a sensation of tension."

In a third class of cases—

The swelling is accompanied by a feeling of smarting, lancinating against the sides in the diseased parts. The skin is hot, shining, and tender, and varies in color from a bright rose-tint to a sombre red. This form, from its clinical history we have named pseudo-phlegmonous.

As we find edema dependent on peripheral nerve lesions, so we find edema due to disease of the cerebro-spinal axis.

This is frequently seen in paralysed limbs after hemiplegia, and in acute myelitis. Vulpian described it as appearing in epidemic cerebro-spinal meningitis. We ourselves have seen it in infantile paralysis. This edema is by no means dependent upon the paralysis, although it frequently accompanies it. In cases of tabes, edema has been observed to appear where there was no paralysis, but when there was a crisis of pain.

We have called attention to this subject, as it is one of great importance and, unfortunately, but little noted.

